# Single-cell analysis of diverse immune phenotypes in malignant pleural effusion

**DOI:** 10.1038/s41467-021-27026-9

**Published:** 2021-11-18

**Authors:** Zhong-Yin Huang, Ming-Ming Shao, Jian-Chu Zhang, Feng-Shuang Yi, Juan Du, Qiong Zhou, Feng-Yao Wu, Sha Li, Wei Li, Xian-Zhen Huang, Kan Zhai, Huan-Zhong Shi

**Affiliations:** 1 Department of Respiratory and Critical Care Medicine, Beijing Institute of Respiratory Medicine and Beijing Chao-Yang Hospital, Capital Medical University, 100020 Beijing, China; 2 Department of Respiratory and Critical Care Medicine, Union Hospital, Tongji Medical College, Huazhong University of Science and Technology, 430022 Wuhan, China; 3 Department of Tuberculosis, Nanning Fourth People’s Hospital, 530022 Nanning, China

**Keywords:** Cancer microenvironment, Non-small-cell lung cancer, Lymphocyte differentiation, Immunosurveillance

## Abstract

The complex interactions among different immune cells have important functions in the development of malignant pleural effusion (MPE). Here we perform single-cell RNA sequencing on 62,382 cells from MPE patients induced by non-small cell lung cancer to describe the composition, lineage, and functional states of infiltrating immune cells in MPE. Immune cells in MPE display a number of transcriptional signatures enriched for regulatory T cells, B cells, macrophages, and dendritic cells compared to corresponding counterparts in blood. Helper T, cytotoxic T, regulatory T, and T follicular helper cells express multiple immune checkpoints or costimulatory molecules. Cell-cell interaction analysis identifies regulatory B cells with more interactions with CD4^+^ T cells compared to CD8^+^ T cells. Macrophages are transcriptionally heterogeneous and conform to M2 polarization characteristics. In addition, immune cells in MPE show the general up-regulation of glycolytic pathways associated with the hypoxic microenvironment. These findings show a detailed atlas of immune cells in human MPE and enhance the understanding of potential diagnostic and therapeutic targets in advanced non-small cell lung cancer.

## Abstract (web summary)

Detailed immune cell subset identification in human lung (malignant pleural effusion) MPE is important for diagnosis and prognosis. Here the authors use single cell RNA sequencing to identify immune cell types within MPE and show differential gene expression compared to circulating immune cells in blood.

## Introduction

Malignant pleural effusion (MPE) is a common and disabling complication of cancer; it accounts for >125,000 hospital admissions per year in the United States^1,2^. The presence of MPE always indicates disseminated or advanced cancer; consequently, the survival is poor, ranging from a median of 3–12 months depending on tumor factors and individual patients^3–5^. It has been documented that the formation of MPE is dictated by a complex tumor–host interplay that triggers pleural inflammation, tumor angiogenesis, and vascular hyperpermeability^6,7^. A large number of immune cells, including lymphocytes and myeloid cells, are enriched in MPE, with CD4^+^ T cells being the dominant cell type^8,9^. Previous studies have demonstrated that multiple subsets of helper T (Th) cells, including regulatory T (Treg), Th1, Th17, Th9, and Th22 cells, as well as activated naive B cells, play important roles in the pathogenesis of MPE^10–15^.

By using single-cell RNA sequencing (scRNA-seq) technique, the landscape of infiltrating immune cells has been demonstrated in non-small cell lung cancer (NSCLC)^16,17^, hepatocellular carcinoma^18,19^, head and neck squamous cell carcinoma^20^, breast cancer^21,22^, and other tumors. These studies have shown that the activation of B cells, increase in Treg cells, decrease in cytotoxic T cells, and the enrichment of exhausted T cells are the immune characteristics of the solid tumor microenvironment. However, most of these studies have been limited to early-stage primary tumors, and specific aspects of MPE-associated tumor microenvironments remain unknown. The major objective of this study was to determine the specific cellular and transcriptional features in MPE at the resolution of single cells using scRNA-seq.

In this work, we show changes in transcriptional state, regulatory networks, and intercellular communication between MPE and matched peripheral blood from the same patients. This study identifies the cellular and biological features that are specific to MPE and describes the different immune status from the primary tumor.

## Results

### scRNA-seq resolves immune cell types in MPE

**Fig. 1:**
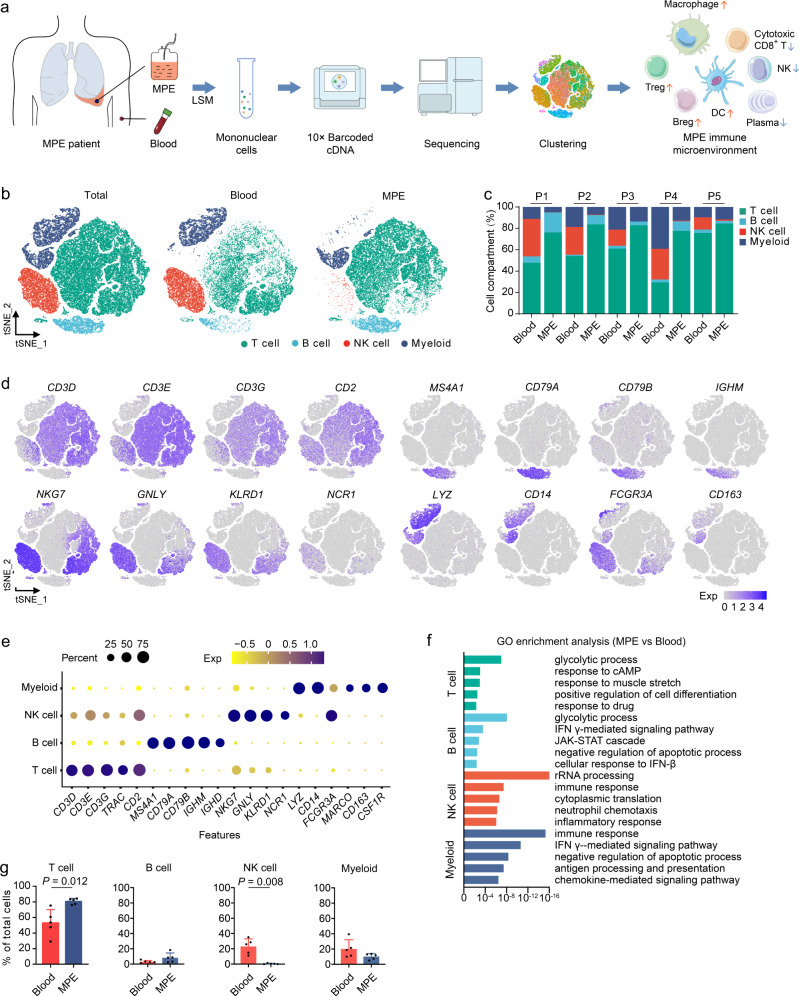
Comprehensive dissection and clustering of single cells from MPE and blood. **a** The flowchart of the overall study design. scRNA-seq and expression analysis of malignant pleural effusion (MPE) and blood samples (*n* = 5) were performed on the 10× Genomics platform. **b** t-SNE plots within each sample type, color-coded by cell types. **c** Average proportion of each cell type derived from each patient, color-coded by cell types. **d** Canonical marker genes for the immune cell types defined in Fig. 1b. Data are colored according to expression levels. **e** Dot plot of average expression of canonical marker genes for the immune cell types defined in Fig. 1b. **f** Gene ontology (GO) enrichment analysis using the genes upregulated in MPE compared with blood for each cell type. The statistical significance was tested by Fisher’s exact test and adjusted by Benjamini–Hochberg correction. **g** Frequencies of four immune cell types in MPE and blood according to the t-SNE plot using scRNA-seq data. Data are presented as mean ± SD. Comparisons were made using two tailed paired Student’s *t* test. Blood, *n* = 5 samples, MPE, *n* = 5 samples.

To characterize the immunological features of MPE environment, we performed droplet-based scRNA-seq to study the transcriptomic profiles of immune cells in MPE and blood (Fig. 1a). Given that neutrophils are the predominant type of leukocytes in blood, whereas lymphocytes are the predominant type of immune cells in MPE, we excluded polynuclear cells from our scRNA-seq analysis and focused on mononuclear cells. After quality filtering of gene expression normalization for read depth and mitochondrial read count, 62,382 cells in which 200–6,000 genes could be detected remained for subsequent analysis. Of these, 33,089 and 29,293 cells originated from MPE and blood, respectively (Fig. 1b and Supplementary Table 1).

We applied principal component analysis across all cells and classified them into 19 groups of cell types using graph-based clustering on the informative principal components (Supplementary Fig. 1a, b). The 19 expression groups were grouped by hierarchical clustering, leading to the characterization of major cellular compartments of T cells, B cells, natural killer (NK) cells, and myeloid cells, with the most abundant MPE immune cells being T cells (Fig. 1c–e and Supplementary Fig. 1c). All cell types were from multiple patients, suggesting that cells were grouped according to the immune-associated characteristics rather than by patient specificity.

For the differentially expressed genes of each cell type between MPE and blood (Supplementary Data 1), we used gene set enrichment analysis and weighed the gene effect on the gene ontology to define pathways related to MPE; we found that the pathways of glycolysis, as well cell proliferation and immune response signals, were significantly higher in MPE (Fig. 1f and Supplementary Fig. 1d). Among the immune cell types, T cells were significantly enriched in MPE, while NK cells were significantly enriched in blood (Fig. 1g).

### Dissection and clustering of T cells in MPE patients

**Fig. 2:**
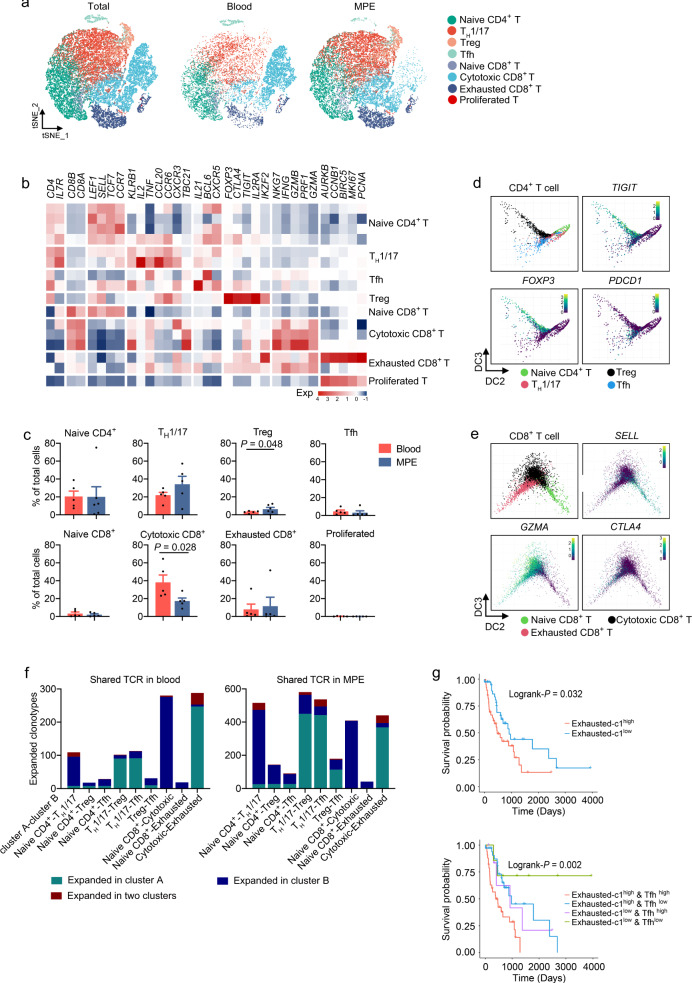
Dissection and clustering of T cells in MPE patients. **a** t-SNE plots of T cells within each cell type, color-coded by T cell subsets. **b** Heatmap of selected T cell marker genes in each T cell subset. **c** Frequencies of T cell subsets in MPE and blood according to the t-SNE plot using scRNA-seq data. Data are presented as mean ± SD. Comparisons were made using two-tailed paired Student’s *t* test. Blood, *n* = 5 samples; MPE, *n* = 5 samples. **d** Diffusion map of CD8^+^ T cell functional state transitions. DC diffusion component. **e** Diffusion map of CD4^+^ T cell functional state transitions. DC diffusion component. **f** Bar plots summarizing the distributions of expanded TCRs between given two clusters. TCR T cell receptor. **g** The Kaplan–Meier overall survival curves of TCGA LUAD patients grouped by the gene signature of exhausted-c1 (upper panel) or exhausted-c1 and Tfh (lower panel). The high and low groups are divided by the median value of the mean expression of signature gene after normalization by T cell fractions estimated by CIBERSORT. The statistical significance was calculated using two-sided log-rank test.

The T cell subsets according to cell lineage and functional state were identified as CD4^+^ T cells (naive CD4^+^ T cells, Th1/17 cells, T follicular helper [Tfh] cells, and Treg cells); CD8^+^ T cells (naive T cells, cytotoxic T cells, and exhausted T cells); and proliferated T cells, which had both CD4^+^ and CD8^+^ T cells (Fig. 2a, b and Supplementary Fig. 2a–d). In accordance with previous findings in NSCLC^16–18^, we also found the depletion of cytotoxic T cells and the emergence of Treg cells in MPE compared with blood (Fig. 2c).

To understand the state transitions among T cell subtypes, we applied Destiny to draw diffusion maps so as to construct the potential developmental trajectories of T cell subtypes based on the expression data. The inferred developmental trajectory from the expression data or marker genes suggested that naive T cells eventually entered a state of exhaustion through cytotoxic T cells in CD8^+^ T cells (Fig. 2d), which is consistent with the previous studies^17^. In CD4^+^ T cells, naive T cells were positioned at the opposite end from Tfh cells and Treg cells, and Th1/17 cells were mainly located at the center (Fig. 2e). *PDCD1*, an important immune-checkpoint target, was highly expressed in Tfh, suggesting that Tfh cells might also be one of the effector cells of PD1 inhibitors in MPE. We next analyzed the transcript difference between Tfh and Treg cells; we found that metabolism-related genes *TPI1*, *FTL1*, *FTH1*, *PKM*, *GPI*, and *SCL16A3* were in the top 20 differentially expressed genes in Tfh cells, indicating that Tfh cells may have unique metabolic characteristics (Supplementary Fig. 2e).

A total of 79.7% CD4^+^ T cells expressed αβ T cell receptor (TCR), and the fraction in CD8^+^ T cells was 78.5%. TCR clonotypes resided both in MPE and in the corresponding blood from the given patient (Supplementary Fig. 2f). Among CD8^+^ T cells, significantly shared TCRs occurred between cytotoxic and exhausted T cells (Fig. 2f). These shared TCRs were found in each sample, which indicates that T cell expansion is a common phenomenon in MPE, rather than a characteristic of certain cancer patients. We labeled these exhausted T cells expressing shared TCR with cytotoxic T cells as exhausted-c1 clusters and the unexpanded exhausted T cells as c2. Exhausted-c1 T cells accounted for 88.8% of the total exhausted T cells; they expressed a higher exhausted signature compared with other cell types (Supplementary Fig. 2g, h). Patients with high exhausted-c1 signature gene expression (after normalizing for cell fractions by CIBERSORT) showed significantly poorer overall survival (Log-rank *P* = 0.032) compared with those with a low expression. Furthermore, we showed that the combination with Tfh signature, which was the exhausted cluster in CD4^+^ T cells, was able to discriminate the prognosis of patients with high exhausted-c1 signature (Fig. 2g). Although further studies are needed, the association of expanded exhausted T cells and unfavorable clinical features may guide future diagnostic strategies and treatment.

### Dissection and clustering of B cells in MPE patients

**Fig. 3:**
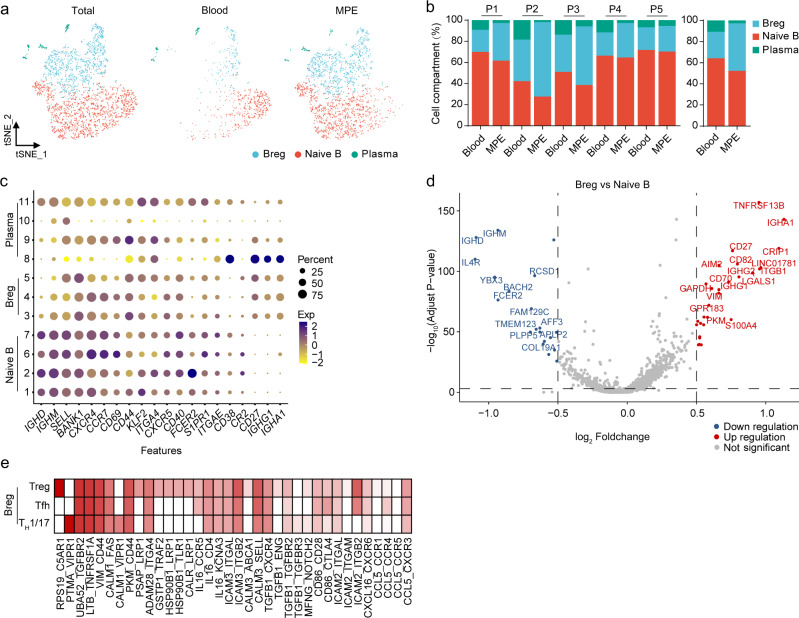
Dissection and clustering of B cells in MPE patients. **a** t-SNE plots of B cells within each cell type, color-coded by B cell subsets. **b** Average proportion of each cell subset derived from each patient (left panel) and MPE or blood (right panel). **c** Dot plot of the average expression of canonical marker genes for B cells. *Y*-axis: Seurat-clusters in Supplementary Fig. 3a. **d** Scatter plot of differentially expressed genes of the Breg cells in comparison with naive B cells in MPE. **e** Heatmap of cell-to-cell interaction scores between Breg cells and Th1/17 cells, Treg cells, or Tfh cells.

We identified seven subpopulations of CD19^+^ B cells and four plasma cell subsets according to the expression and distribution of canonical B cell markers^23^. Four B cell subsets highly expressed *IGHD*, *TCL1A*, and *IL4R*—the hallmark genes of naive cells—and showed low-to-no expression of *CD27*; these B cell subsets were designated as naive B cells. Three B cell subsets highly expressed *CD24* and *CD27* and were designated as regulatory B (Breg) cells. Plasma B cells expressed immunoglobulin gamma (*IGHG*) and *XBP1* (Fig. 3a–c and Supplementary Fig. 3a–c). Breg cells were significantly enriched in MPE, while plasma cells were significantly enriched in blood (Supplementary Fig. 3d).

To further investigate differential transcriptomic changes in B cells, we compared the expression profiles between Breg cells and naive B cells in MPE. We compared the differential genes of naive B cells and Bregs in MPE and blood, respectively. We concluded that top-ranked upregulation of gene expression in naive B cells and Bregs in MPE was in a similar enrichment pattern. Both naive B and Breg cells in MPE expressed more glucose metabolism (*PKM*, *TPI1*, *ENO1*, and *LDHA*), hypoxia response (*NR4A2*, *CXCR4*, and *HIF1A*), and cell proliferation (*H3F3B*, *MIF*, and *ZFP36L2*) signaling compared with their corresponding counterparts in blood. *TNFRSF13B*, *ITGB1*, and *LGALS1* genes were all significantly highly expressed in Breg cells (Fig. 3d), indicating that Breg cells have the function of regulating other important immune cells, such as T cells, through cell-to-cell interaction. To explore the interaction between Breg cells and T cells, we examined whether specific ligand–receptor pairs could inform the interaction between Breg cells and effector T cell subsets (Th1/17 cells, Treg cells, Tfh cells, cytotoxic T cells, and exhausted T cells) that drives the adaptive immunity network. Our data showed that, in MPE, the number of predicted interactions between Breg cells and CD8^+^ T cells (both cytotoxic and exhausted T cells) was strongly reduced, and the cell–cell communication landscape of Breg cells was dominated by CD4^+^ T cells, including Th1/17, Treg, and Tfh (Fig. 3e and Supplementary Fig. 3g).

B cell receptor (BCR) was detected on the surface of 91.4% of B cells. We first performed B cell Ig isotype analyses. A total of 85.4% of naive B cells expressed *IgM*, and *IgD* was expressed in the rest. The proportion of *IgM* expressed in Breg cells was <35%, and the proportion of *IgG* expression was 41.9%. Almost no *IgE*^+^ B cells were detected. Both in total B cells and in three B cell clusters, the expression ratios of the four Ig isotypes were not different between MPE and blood (Supplementary Fig. 4a, b). No significant BCR expansion was found in B cells either in MPE or in blood, indicating that B cell immune response to tumor might not be from B cell clonotypic expansion (Supplementary Fig. 4c).

### Dissection and clustering of myeloid cells in MPE patients

**Fig. 4:**
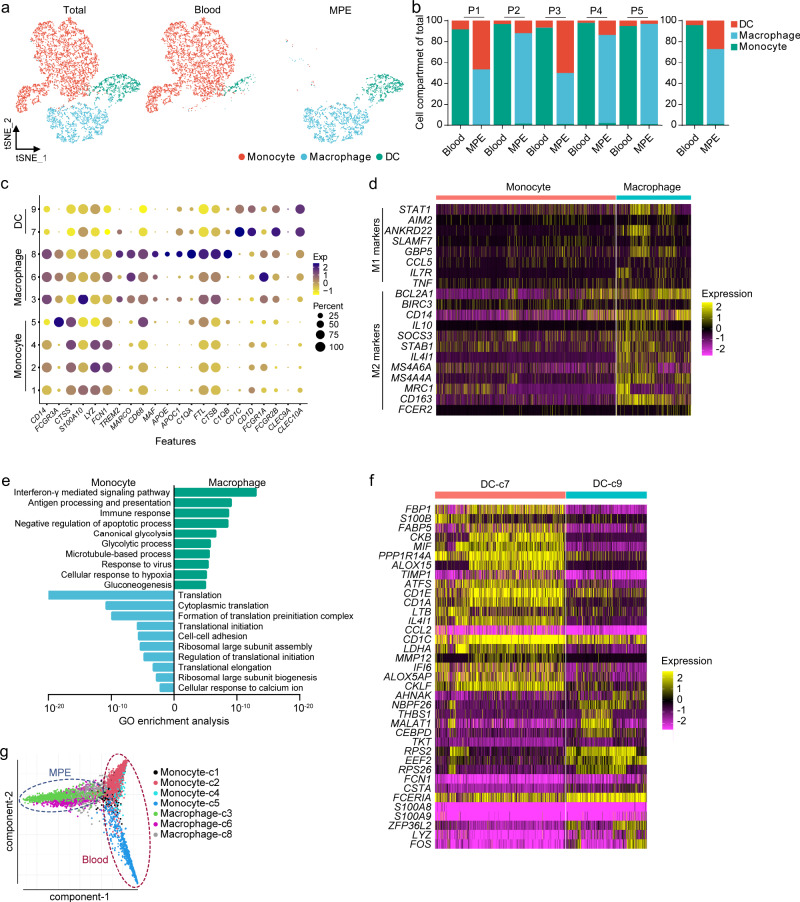
Dissection and clustering of myeloid cells in MPE patients. **a** t-SNE plots of myeloid cells within each cell type, color-coded by myeloid cell subsets. **b** Average proportion of each cell subset derived from each patient (left panel) and MPE or blood (right panel). **c** Dot plot of the average expression of canonical marker genes for myeloid cells. *Y*-axis: Seurat-clusters in Supplementary Fig. 4a. **d** The heatmap of M1 and M2 marker genes in monocytes or macrophages. **e** GO enrichment analysis between monocytes and macrophages. **f** The heatmaps show the genes differentially expressed in dendritic cell (DC) subsets. The statistical significance was tested by Fisher’s exact test and adjusted by Benjamini–Hochberg correction. **g** Diffusion map of myeloid cell subset functional state transitions.

We detected canonical marker genes of myeloid cells and identified clusters in MPE and blood. Seven monocyte/macrophage clusters were defined by high expression levels of marker genes *CD14*, *FCGR3A*, and *CD68*. Two dendritic cell (DC) clusters were designated by high expression levels of *CD1C*, *CLEC10A*, and *FCGR1A* (Fig. 4a–c and Supplementary Fig. 5a–c).

Macrophages were significantly enriched in MPE and divided into three subsets, and monocytes were enriched in blood and were divided into four subsets (Supplementary Fig. 5d). Macrophage phenotypes are divided into M1 or M2 subsets with antitumorigenic and protumorigenic functions, respectively^24^. We examined the expression levels of marker genes for M1 (e.g., *STAT1* and *CCL5*) and M2 (e.g., *MRC1*, *MS4A4A*, *IL4I1*, and *CD163*) states across the macrophage clusters and found that MPE macrophages highly expressed M2 marker genes (Fig. 4d). This indicates that the M1/M2 classification had no obvious relationship with the subclasses of monocyte/macrophages based on gene expression. The immune response, antigen presentation, and glycolysis pathway activity of macrophages were significantly enhanced as compared with monocytes (Fig. 4e). DCs had two subsets; DC-c7 was significantly enriched in MPE and had higher levels of immunoactivated genes *MIF*, *ALOX5*, *CKLF*, and *CD1s* (Fig. 4f). The activated DC cluster was enriched not only in genes encoding antigen presentation but also in genes of glycolysis (Supplementary Fig. 5e). We analyzed the trajectory of MPE macrophages and blood monocytes; we found that these cells have their own trajectory states, suggesting that tumor-infiltrating macrophages are different from monocytes (Fig. 4g).

### Cell-type-specific metabolic reprogramming

Our data showed that immune cells exhibited different metabolic transcriptome characteristics, among which Treg cells, Tfh cells, cytotoxic CD8^+^ T cells, exhausted CD8^+^ T cells, and NK cells had a higher overall expression of metabolism-related genes, compared with other cell types (Supplementary Fig. 6a). Such observations suggested that these immune effector cells had higher metabolic demands.

**Fig. 5:**
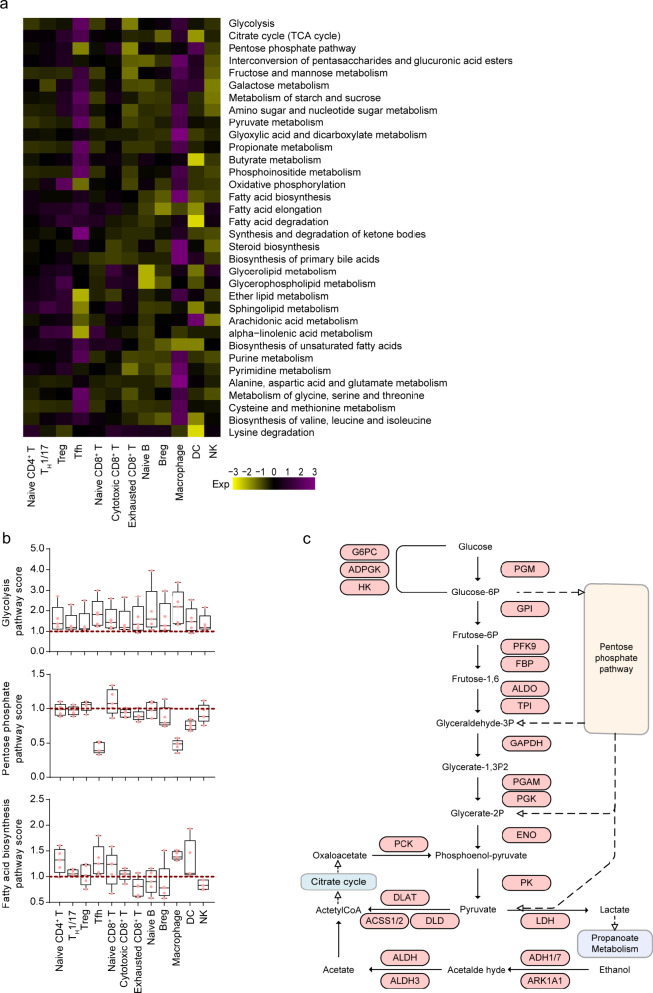
Metabolic heterogeneity in MPE patients. **a** Heatmap of the indicated metabolic pathway scores in MPE. The expression value used in the heatmap is the ratio of the average enrichment scores of the corresponding pathway in MPE and blood. **b** Distributions of the average enrichment score of the indicated metabolic pathways in MPE immune cell subtypes. **c** Map of glycolysis metabolic pathways of Tfh cells. The genes marked in red are upregulated in MPE vs. blood. *n* = 5 samples. The box plots were defined by the interquartile range (IQR, the range between the 25% and 75%) and the median, whiskers represent the upper and lower value within 1.5 times the IQR.

Compared with blood, the most increased expression of the metabolic pathways was found in Tfh and macrophages in MPE (Fig. 5a). Nearly all cell types in MPE upregulated the expression of metabolic genes involved in glycolysis; this universal metabolic feature of these immune cell subtypes was in line with the hypoxic environment in pleural effusion (Fig. 5b). Interestingly, compared with cells in the blood, Tfh cells in MPE exhibited significant upregulation of glycolysis, cysteine, and methionine metabolism in addition to a decrease in the pentose phosphate pathway, arachidonic acid metabolism, and alpha-linolenic acid metabolism (Supplementary Fig. 6a, b). In the glycolytic metabolism pathway map drawn with the highly expressed genes in MPE by Tfh cells, we noticed some rate-limiting genes, such as *HK*, *PFK*, and *PK*, thereby further confirming that glycolytic metabolism of Tfh cells is activated in MPE (Fig. 5c). In addition, pentose phosphate pathway was significantly downregulated, whereas OXPHOS and fatty acid oxidation were slightly upregulated, which is consistent with M2 polarization phenotype of macrophages in MPE (Fig. 5b and Supplementary Fig. 6b). Taken together, these metabolic phenotypes of immune cells may help to establish their functions in interacting with other cell types and modulating the tumor microenvironment.

### Cluster-specific expression of genes associated with disease risk

**Fig. 6:**
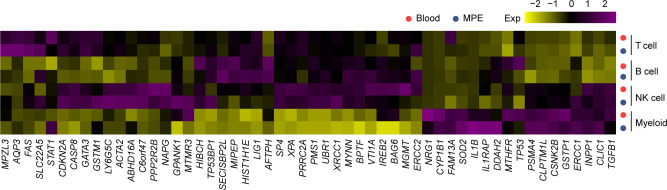
The expression of genes determined by genome-wide association studies in MPE and blood. The heatmaps show the average expression levels of genes previously indicated in genome-wide association studies of non-small cell lung cancer in Chinese Han.

Previously, genome-wide association studied (GWASs) have identified 54 genes associated with susceptibility to NSCLC in the Chinese population^25–28^. We further analyzed the expression levels of these genes across the clusters identified in MPE patients and found both expected and surprising cluster-specific expression patterns (Fig. 6). *BPTF* was highly expressed in NK cells and T cells, inhibiting NK cell activity and reducing T cell-mediated antitumor immunity^29,30^. Among the subgroups of T cells, *BPTF* showed the highest expression in Treg cells and exhausted CD8^+^ T cells (Supplementary Fig. 7a). Specifically, the expression of *BPTF* in MPE was significantly higher than that in blood, consistent with the exhaustion state of immune cells in MPE. *STAT1*, which participates in interferon (IFN) immune regulation of B cells^31^, was highly expressed in all B cell subtypes in MPE (Supplementary Fig. 7b). *IL1B* was expressed in myeloid cells (Supplementary Fig. 7c); it is an important mediator of the inflammatory response involved in a variety of cellular activities, including cell proliferation, differentiation, and apoptosis^32^. Some susceptibility genes lacking functional research in tumors, including *AFTPH*, *PRRC2A*, *HIST1H1E*, and *MIPEP*, were also highly expressed in immune cells. Our results also showed that there were changes in the expression of susceptibility genes in the immune cells of tumor patients, suggesting that these single-nucleotide polymorphisms and susceptibility genes might affect the occurrence and development of tumors by regulating the function of immune cells.

### Chemokine signature in macrophages is associated with lung adenocarcinoma (LUAD) prognosis

**Fig. 7:**
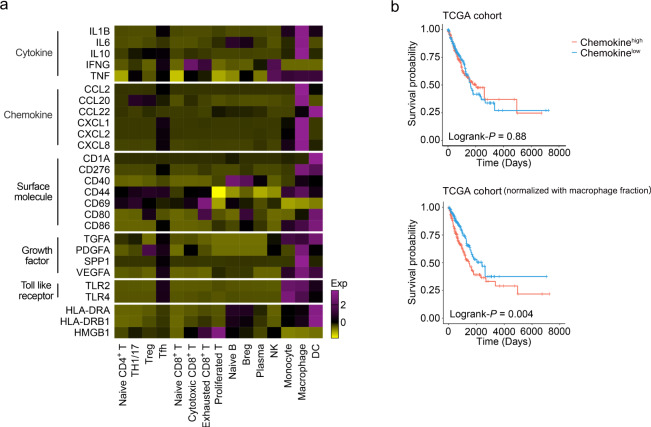
Expression and survival analysis of genes associated with MPE formation. **a** The heatmap shows the mean expression of genes previously indicated to be associated with MPE formation. **b** The Kaplan–Meier overall survival curves of TCGA LUAD patients grouped by the gene signature of chemokine. The high and low groups are divided by the median value of mean expression of chemokine (upper panel) or with normalization by CIBERSORT (lower panel). Statistical significance was calculated using two-sided log-rank test.

Next, we assessed the contribution of specific immune cell types to MPE formation. In the pleural cavity, lymphocytes govern the occurrence of vasoactive events and the development of an MPE by important signal molecules, such as chemokines, cytokines, and growth factors^6,33,34^. Previous studies have revealed several signaling pathways involved in MPE formation. We considered the generality and prognostic significance of the key signaling molecules in individual cellular components. Chemokine signature (including *CCL2*, *CCL20*, *CCL22*, *CXCL1*, *CXCL2*, and *CXCL8*), with a higher expression in macrophages, had no association with the survival of LUAD patients in the Cancer Genome Atlas (TCGA) cohort. However, after normalization for macrophage fractions, patients with high expression of the chemokine signature showed significantly poorer overall survival compared with those with a low expression (Fig. 7). Although several molecular mechanism studies on these important signaling pathways have been investigated in MPE formation, their associations with MPE diagnosis and therapy are still controversial. Our findings provided support for the association of cell-specific expression of chemokine signatures with the prognosis.

## Discussion

In the present study, we provided a detailed molecular description of immune cells in human MPE at the single-cell level, charting differences in frequencies and molecular state of immune cells in MPE and blood. Specifically, we noted the increases in the numbers of Treg cells, B cells, and macrophages in MPE and showed transcriptional cell states important to MPE, mainly including Tfh cells and Breg cells. Moreover, we identified Breg cell-specific intercellular communication with CD4^+^ T cells in the MPE environment. We discovered that glycolytic pathways are generally upregulated in MPE immune cells.

By using scRNA-seq, Lavin et al. demonstrated that, as early as in stage I, NSCLC lesions have a strongly reduced CD8^+^ T effector/Treg ratio compared with normal tissues^16^; Zhang et al. demonstrated that the state of CD8^+^ T cells in primary NSCLC appears to be shaped by two distinct processes, the inherent T cell developmental program and the tumor-induced T cell exhaustion^8^. The expression of T cell inhibitory receptors is associated with T cell dysfunction in the tumor environment. Consistent with previous research^35^, T cells expressed a higher level of inhibitory receptors in MPE compared with T cells in blood. Moreover, Tfh and exhausted-c1 cells expressed the highest levels of inhibitory receptors in CD4^+^ T and CD8^+^ T cells, respectively. We identified the cell cluster with the highest exhausted score among CD4^+^ and CD8^+^ T cells in the MPE immune microenvironment.

Immunosuppressive signals play important roles in cancer progression. Previously, we have shown that, compared with pleural lavage from lung cancer patients without MPE, the numbers of Treg cells in MPE from lung cancer patients were significantly increased, and these Treg cells interact with other Th cells and play immunosuppressive roles in the pathogenesis of MPE^10–14^. By using scRNA-seq in the present study, we further found that naive T cells differentiated into Treg cells and Tfh cells through the intermediate state of Treg cells and that Tfh cells expressed higher levels of immunosuppressive signals compared with Treg cells (Supplementary Fig. 2h). In addition, CD16^+^ NK cells were strongly reduced in MPE, and the pleural NK cells expressed poorly cytolytic, and expansion or activation, signaling, including lower levels of granzyme B and less IFN-γ, compared with blood NK cells. These findings regarding Tfh and NK cells in MPE might provide insights into the mechanisms of tumor immunosuppression in advanced NSCLC.

Compared with T cell subsets, knowledge about the functions of B cell subsets in MPE progression is still insufficient. Previous studies have shown a mechanism by which activated naive B cells promote MPE formation by regulating Th1/Th17 cell responses^15^. In line with previous findings, naive B cells were less abundant in MPE than in blood. More importantly, we identified three Breg cell subsets, and all three Breg cell subsets were increased in MPE. Furthermore, we found that both naive B cells and Bregs showed strong signals of glycolytic, hypoxia response, and IFN-γ signaling pathways in MPE compared with blood; we also identified that the interaction between Bregs and T cells mainly occurred in CD4^+^ T cells, instead of CD8^+^ T cells.

It has been shown that the enrichment of macrophage gene signatures is significantly associated with a survival disadvantage in lung cancer^36,37^. Previous study has further demonstrated that M2 macrophages are remarkably more common in MPE than in benign pleural effusion and that the number of M2 macrophages is a helpful index for differential diagnosis of MPE^38^. A previous study reported that MPE-Mφ had both M1 and M2 macrophage gene expression patterns^39^; likewise, both M1 and M2 signatures were recorded in macrophages in our sequencing data, but obviously, the M2 signature was stronger than that of M1. Looking for an efficient way to reverse the signature of macrophages and increase the M1 macrophage population might become a direction for tumor therapy. As expected, macrophages in MPE had distinct cellular and gene expression changes as compared with monocytes in blood; moreover, MPE macrophages highly expressed M2 marker genes, suggesting M2 macrophages as a potential cellular candidate for therapeutic target of MPE.

Tumor cells undergo metabolic changes in order to adapt to the tumor microenvironment, and immune cells require complex metabolic transcriptome heterogeneity to perform their specific functions. In MPE, several important immune cells, including Treg cells, Tfh cells, cytotoxic CD8^+^ T cells, exhausted CD8^+^ T cells, and NK cells, exhibit a higher overall expression of metabolic transcriptome characteristics. Specifically, compared with their counterparts in blood, Tfh cells in MPE exhibited the significant upregulation of glycolysis, cysteine, and methionine metabolism in addition to the decrease in the pentose phosphate pathway, arachidonic acid metabolism, and alpha-linolenic acid metabolism. Pathway mapping of genes involved in glycolytic metabolism confirmed that MPE Tfh cells upregulated the expression levels of rate-limiting steps in the glycolytic metabolic pathway, such as *HK*, *PFK*, and *PK*.

Although previous studies have shown that CD4^+^ T cells exhibited significantly higher levels of OXPHOS compared with CD8^+^ T cells^40^, similar changes were not observed in our study. Previous studies have also shown that hypoxia activates signal transduction pathways that induce glycolysis and suppress mitochondria-associated pathways^41,42^; therefore, the interaction between hypoxia, glycolysis, and energy metabolism is highly dynamic in MPE cells, and the quantitative relationship between them is at least partially determined by the inhibitory effect of the hypoxia-inducible factor signaling pathway on oxygen availability. However, our finding of several immune cells with increasing glycolytic activity in MPE might be a consequence of more severe hypoxic environment in MPE than in primary lung tumor or blood, and these cells may consume more energy to support their cytotoxic capacity and function in tumor immunity.

By comparing the gene expression profiles of single cells from MPE and blood, we were able to identify metabolic features among different subpopulations of immune cells whose metabolism was greatly influenced by the shortage of nutrients in MPE. We found that some cell subpopulations in MPE, including Tfh cells, cytotoxic CD8^+^ T cells, and macrophages, adopted metabolic phenotypes distinct from those in blood. These results highlight the tremendous impact of the microenvironment on cellular metabolism. The differences in the environment cause the metabolic features of subpopulations of immune cells in MPE to be different from those in blood, possibly also in primary tumors.

We investigated the relationship between the signature genes of 15 cell clusters identified in MPE and patient survival in all TCGA LUAD cases or advanced cases (stage III and IV) and found no signature gene set associated with the patients’ survival (Supplementary Fig. 8). In addition, we linked the pathologic stage with cellular subpopulation fraction in the TCGA cohort. Twenty-two immune cell fractions identified by CIBERSORT were used as control. We used the marker gene expression profiles in the MPE data and in the CIBERSORT algorithm to calculate the proportion of macrophages in TCGA LUAD. The proportions and the trend of changes with the stage of macrophage calculated by these two data sets were consistent, indicating that the cell proportion of TCGA data calculation using our expression profile is reliable. The ratio of Th1/17 decreased and the ratio of exhausted T cells increased, suggesting that these two cell types might be linked to tumor progression (Supplementary Fig. 9). Furthermore, combining the results of TCR sequencing, the exhausted CD8^+^ T cells were divided into the expanded c1 cluster and the non-expanded c2 cluster. Patients with high exhausted-c1 signature gene expression (after normalizing for cell fractions by CIBERSORT) showed significantly poorer overall survival compared with those with a low expression. Furthermore, we showed that, combined with the Tfh signature, which was the exhausted cluster in CD4^+^ T cells, the exhausted signature was able to discriminate the prognosis of patients with high exhausted-c1 signature (Fig. 2g). Taken together, we identified T cell clusters, expanded exhausted T cells, predicting poor prognosis in advanced LUAD, and the statistical results were improved when combined with Tfh cells. Signature genes of expanded exhausted T cells and Tfh cells might serve as potential clinical biomarkers for advanced LUAD patients.

In conclusion, this study offers a global picture of immune cells from the complex MPE microenvironment and depicts transcription feature activities of immune cells that are distinct from those of blood immune cells. Our data can be used as a resource for follow-up in-depth research to complete deeper biological exploration and provide therapeutic targets and biomarkers for the immunotherapy of advanced NSCLC.

## Methods

### Study patients and sample collection

This study was conducted in accordance with the approved guidelines of the Institutional Review Boards of Beijing Chao-Yang Hospital, Capital Medical University; Union Hospital, Tongji Medical College; and Nanning Fourth People’s Hospital. Five patients with definite diagnosis of MPE (4 men and 1 woman; mean age, 65 years [range, 56‒72 years]) were enrolled in our study (Supplementary Table 1), and all the study participants provided written informed consent. Pleural fluid samples from each subject were collected in heparin-treated tubes within 24 h after hospitalization, using standard thoracentesis techniques. Approximately 5 mL venous blood was drawn simultaneously. The pleural effusion (PE) specimens were immediately immersed in ice and were then centrifuged at 800 × *g* for 5 min. The cell-free supernatants of PE and sera were frozen at −80 °C immediately after centrifugation. Mononuclear cells from pleural fluid and blood were isolated by Ficoll-Hypaque gradient centrifugation (Pharmacia, Uppsala, Sweden) within 1 h and were resuspended in phosphate-buffered saline.

### Single-cell cDNA library preparation and sequencing

In accordance with the manufacturer’s instructions (10× Genomics, Pleasanton, CA), single-cell libraries were constructed using the Single Cell 5′ Library & Gel Bead Kit (1000167), Single Cell V(D)J Enrichment Kit, Human T Cell (1000005), or Single Cell V(D)J Enrichment Kit, Human B Cell (1000016). According to the cDNA synthesis and Chromium Single Cell A Chip Kits (120236, 10× Genomics), cell suspensions (300‒600 living cells per microliter) were loaded on a Chromium Controller (1000202, 10× Genomics) to generate single-cell gel beads in the emulsion. The cDNA quality was assessed using an Agilent 4200 TapeStation System (Agilent, Santa Clara, CA). The cDNA libraries were constructed using the Single Cell 5′ Library Construction Kit (1000020, 10× Genomics) and i7 Multiplex Kit (120262, 10× Genomics) and sequenced using a NovaSeq 6000 System (Illumina, San Diego, CA) with a pair-end 150 bp reading strategy. For each sample, at least 220 GB sequencing data were generated for gene transcriptome sequencing and at least 10 GB of sequencing data were generated for TCR/BCR repertoires (performed by CapitalBio, Beijing, China). The data were finally aligned to the GRCh38.93 reference genome with Cell Ranger (v.3.0.2, 10× Genomics) using “mkfastq” and “count” commands and default parameters. The results of the Cell Ranger analysis contained the count values of unique molecular identifiers assigned to each gene in each of the cells for each individual sample using all mapped reads.

### Cell filtering and quality control

The output filtered gene expression matrices were analyzed by the Seurat software package (v.3.0.0) of R software (v.3.5.3). Genes with an expression ratio of >0.1% and cells with 200–6,000 detected genes were selected for further analyses. Low-quality cells with >10% unique molecular identifiers derived from the mitochondrial genome and doublets identified by DoubletFinder were removed (Supplementary Table 1).

### Cell clustering

Seurat was used to complete the clustering of immune cells. We used the “NormalizeData” function to normalize and homogenize the data with the default scaling parameter of 10,000 and used log1p to perform natural logarithmic conversion. “FindVariableGenes” function was used to identify highly variable genes with parameters for “mean.function = ExpMean, dispersion.function = LogVMR, x.low.cutoff = 0.0125, x.high.cutoff = 3, and y.cutoff = 0.5.” We standardized the data with the “ScaleData” function. After performing PCA analysis using highly variable genes, the first 20 principal components and a resolution of 0.8 were selected for the following cluster analysis and visual dimensionality reduction by *t*-distributed stochastic neighbor embedding. We used the “FindAllMarkers” or “FindMarkers” function to determine the marker genes of each cluster relative to all other clusters or to a specific cluster. The selected parameters of marker genes were detected in at least 30% of the cells in the target cluster, under *P* value of Wilcoxon test <0.05 and the differential expression threshold of 0.25 log fold change. FeaturePlot, DotPlot, VlnPlot, and DoHeatmap were used for visualization of gene expression levels. We labeled the obtained clusters as T cells, B cells, NK cells, or myeloid cells through known classic markers (T cells: *CD3D*, *CD3E*, *CD3G*, *CD2*, *TRAC*; B cells: *MS4A1*, *CD79A*, *CD79B*, I*GHM*, *IGHD*; NK cells: *NKG7*, *GNLY*, *KLRD1*, *NCR1*; myeloid cells: *LYZ*, *CD14*, *FCGR3A*, *CD163*, *CSF1R*) and finally analyzed each of these clusters separately to identify the finer clusters by repeating the above operations.

### Differentially expressed gene and pathway analysis

Expression matrix derived between sets of cells was analyzed by the differentialGeneTest function of Monocle 3 for differential gene analysis. The gene ontology annotation was also performed by Monocle 3 with the compareCluster function (fun = “enrichGO”). Significantly different values were determined with an False Discovery Rate-corrected *P* value < 0.05. For metabolic analysis, metabolism pathway signature genes were downloaded from KEGG (Supplementary Data 2) and the average enrichment score was determined for each cluster.

### TCGA data analysis

The gene expression data and the clinical data were downloaded from cBioPortal (http://www.cbioportal.org/). A total of 406 patients with survival information and no preoperative treatment were included in the follow-up analysis. CIBERSORT algorithm was used to calculate the proportion of immune cell types identified by CIBERSORT or our MPE data^43^. To eliminate the influence of different immune cell proportions, for each gene set, we divided the expression in the TCGA data by the cell fraction estimated by CIBERSORT for normalization. According to the median of the average expression after normalization of the gene set, the patients were divided into a high expression group and a low expression group. The Kaplan–Meier survival curve analysis was performed by “survival” (3.2-10) and “survminer” (0.4.9) packages in R, and it showed the prognostic results between the high- and low-expression groups. *P* < 0.05 was set as the significance threshold.

### Signaling score

AddModuleScore function in the Seurat was used for signaling score calculation. Genes used for exhausted score in CD8^+^ T cells were: *HAVCR2*, *CXCL13*, *CCL3*, *SIRPG*, *IFNG, TIGIT*, *GZMB*, *PDCD1*, *PARK7*, *TNFRSF9*, *ACP5*, *CTLA4*, *RBPJ*, *CXCR6*, *CD27*, *FKBP1A*, *BST2*, *TPI1*, *MIR155HG*, *PTTG1*, *CD63*, *SAMSN1*, *RGS1*, *ITGAE*, *HLA-DRA*, *IGFLR1*, *KRT86*, *ENTPD1*, *DUSP4*, *SIT1*, *TOX*, *PHLDA1*, *CCND2*, *GPR25*, *LAYN*, *PRDX5*, *SARDH*, *FASLG*, *ANXA5*, *CTSD*, *PDIA6*, *RANBP1*, *COTL1*, *TNFRSF1B*, *IDH2*, *CD38*, *CD82*, *LAG3*, *MIR497HG*, *APOBEC3C*, *ITM2A*, *COX5A*, *IFI35*, *NDFIP2*, *TNFRSF18*, *KRT81*, *DNPH1*, *RGS2*, *HMGN1*, *DYNLL1*, *SNRPB*, *SYNGR2*, *RAB27A*, *PSMC3*, *GALM*, *FABP5*, *UBE2L6*, *MYO7A*, *PRDX3*, *DDIT4*, *STMN1*, *CDK2AP2*, *VCAM1*, *SNAP47*, *PSMB3*, *ISG15*, *HLA-DRB5*, *CKS2*, *TNIP3*, *CD7*, *PSMD4*, *ATP6V1C2*, *PSMD8*^17^.

### Trajectory analysis

Destiny software package (v.2.6.2) was used to perform the trajectory analysis based on dimensionality reduction using diffusion maps. In each case, only the cells relevant to the question at hand were analyzed.

### Interactions between cell types

Ligand–receptor interactions were determined based on the expression of ligands by one cell type and corresponding receptors by another cell type. Ligand–receptor interacting pairs were referenced from CellPhoneDB (www.CellPhoneDB.org, accessed on 10/30/2019). The corresponding values were used to identify potential cell–cell interactions and significant interactions. Seventy-three ligand–receptor pairs were identified with at least one comparison with *P* < 0.05 and visualized as a heatmap using R package.

### GWAS-related gene scores

NSCLC-associated GWAS genes are listed in Supplementary Table 2^25–28^. We analyzed the expression patterns of 54 genes previously reported in the GWAS of NSCLC in Chinese Han. For each gene, we calculated its average scaling (*Z* normalized) expression. The average enrichment score of GWAS genes was determined for each cluster and represented as a heatmap.

### TCR and BCR analysis

Cell Ranger was used for alignment, filtering, barcode counting, and UMI counting for immune repertoire analysis. Cells with unique productive TCR a–β pairs or with unique productive BCR H chain were analyzed. The expanded cells were defined as those with the β chain or H chain shared by at least two cells.

### Reporting summary

Further information on research design is available in the Nature Research Reporting Summary linked to this article.

## Supplementary information

Supplementary Information

Description of Additional Supplementary Files

Supplementary Data 1

Supplementary Data 2

Reporting Summary

## Data Availability

The raw sequence data and partially processed data have been deposited in Genome Sequence Archive for Human (HRA000153) and Gene Expression Omnibus database (GSE185058). The remaining data are available within the article and Supplementary Information. Source data are provided with this paper.

## Source data

Source Data

## Code availability

Example scripts to process and analyze data are available at https://github.com/michellecyyy/scRNAseq_MPE. Detailed information is available from the corresponding author upon reasonable request.
