# Supplementary material for: Single-cell analysis of diverse immune phenotypes in malignant pleural effusion
Source: Nat Commun. 2021 Nov 18;12:6690. doi: 10.1038/s41467-021-27026-9 (PMC8602344; doi:10.1038/s41467-021-27026-9)
Supplement: Supplementary file 1 — Supplementary Information [file 41467_2021_27026_MOESM1_ESM.pdf]

Single-cell analysis of diverse immune phenotypes in malignant pleural effusion

Short running head: Immune Cells in Pleural Malignancy

Zhong-Yin Huang[1]†, Ming-Ming Shao[1]†, Jian-Chu Zhang[2]†, Feng-Shuang Yi[1], Juan Du[1], Qiong Zhou[2], Feng-Yao Wu[3], Sha Li[3], Wei Li[3], Xian-Zhen Huang[3], Kan Zhai[1]*, Huan-Zhong Shi[1]*

[1]Department of Respiratory and Critical Care Medicine, Beijing Institute of Respiratory Medicine and Beijing Chao-Yang Hospital, Capital Medical University, Beijing 100020, China; [2]Department of Respiratory and Critical Care Medicine, Union Hospital, Tongji Medical College, Huazhong University of Science and Technology, Wuhan, China; [3]Department of Tuberculosis, Nanning Fourth People's Hospital, Nanning 530022, China

*Corresponding author. Email: shihuanzhong@sina.com (H.Z.S.); zhaikan@ccmu.edu.cn (K.Z.)

†These authors contributed equally to this work.

Supplementary Fig. 1. Single-cell transcriptional profiling of immune cells from MPE and blood.

Supplementary Fig. 2. Single-cell transcriptional profiling of T cells from MPE and blood.

Supplementary Fig. 3. Single-cell transcriptional profiling of B cells from MPE and blood.

Supplementary Fig. 4. Clonal expansion of B cells defined by BCR.

Supplementary Fig. 5. Single-cell transcriptional profiling of myeloid cells from MPE and blood.

Supplementary Fig. 6. Metabolic heterogeneity in MPE and blood.

Supplementary Fig. 7. Cell-type specificity of genes associated with non-small cell lung cancer susceptibility in immune cells from MPE and blood using scRNA-seq.

Supplementary Fig. 8. Survival analysis for signature genes of 15 cell types in TCGA LUAD data.

Supplementary Fig. 9. Proportion of cell types in TCGA LUAD data.

Supplementary Table 1. Metric information for five MPE patients induced by lung adenocarcinoma.

Supplementary Table 2. Gene list in genome-wide association studies related analysis.

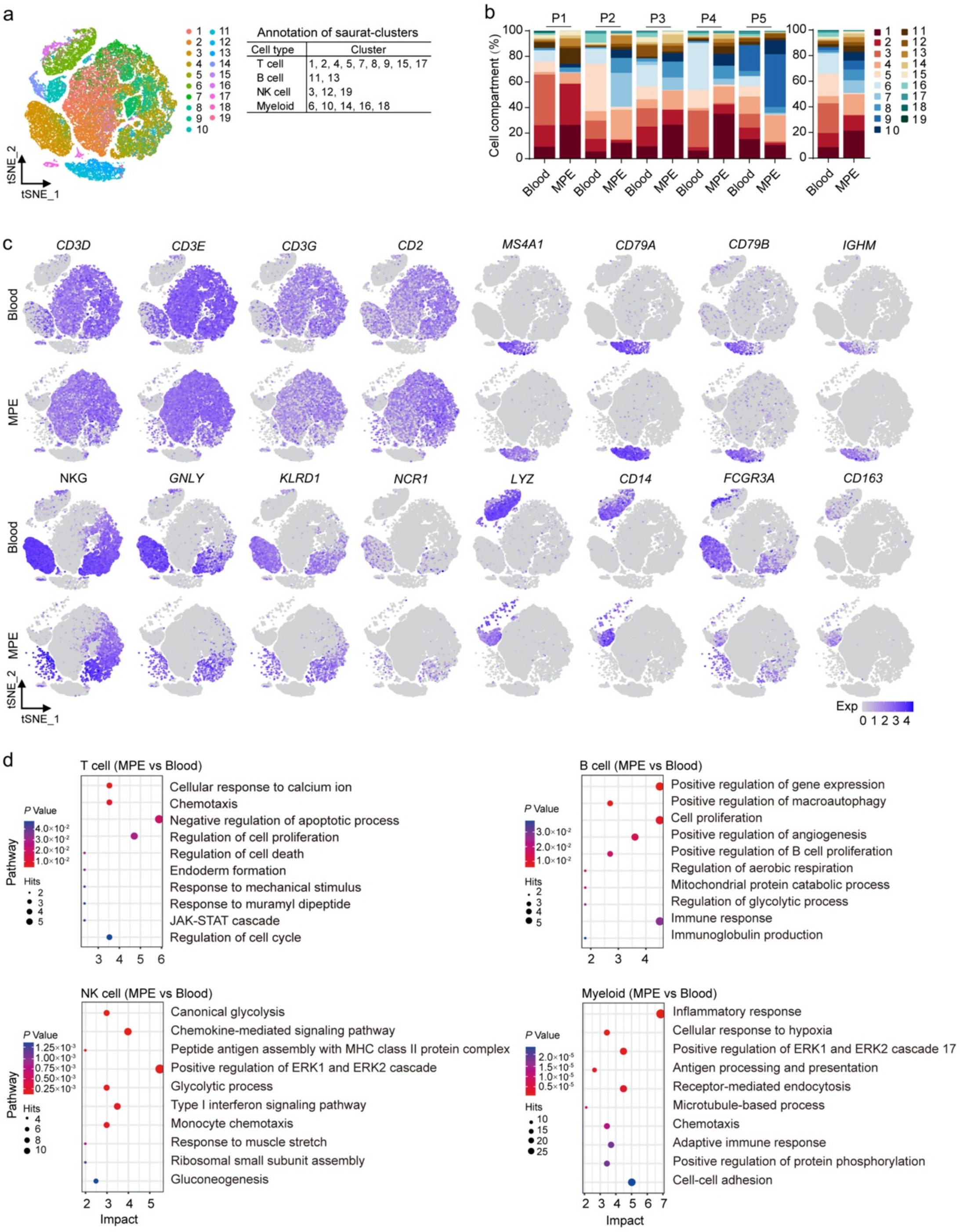

Supplementary Fig. 1. Single-cell transcriptional profiling of immune cells from MPE and blood. a, tSNE plots of total cells and annotation of seurat-clusters with cell types. **b**, Average proportion of each seurat-cluster derived from each patient (left panel), and malignant pleural effusion (MPE) or blood (right panel). **c**, Canonical cell markers for the immune cell types defined in Figure 1b. Data are colored according to expression levels. **d**, Gene Ontology (GO) enrichment analysis using genes upregulated in MPE compared with blood for each cell type. The significance was by Fisher's exact test and adjusted by Benjamini-Hochberg correction.

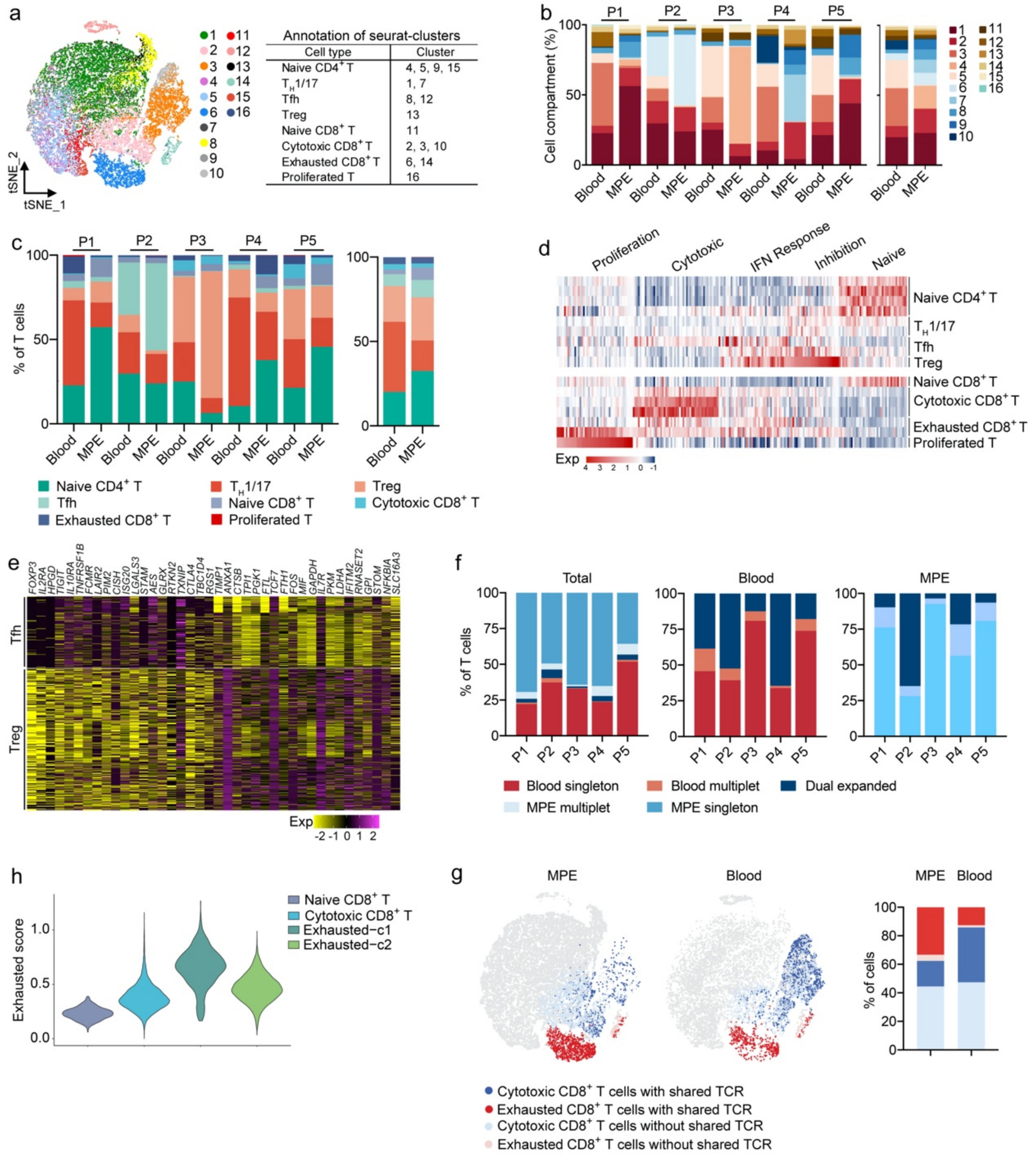

Supplementary Fig. 2. Single-cell transcriptional profiling of T cells from MPE and blood. **a**, tSNE plots of T cells and annotation of seurat-clusters with cell subtypes. **b**, Average proportion of each seurat-cluster derived from each patient (left panel), and MPE or blood (right panel). **c**, Average proportion of each cell subset derived from each patient (left panel), and MPE or blood (right panel). **d**, Heatmap of selected differentially expressed genes in each cell subset. **e**, Heatmap of differently expressed genes of Treg cells in comparison with Tfh cells in MPE. **f**, Fraction of the clonotype fraction and cell fraction. Bar plot shows the clonotype fraction in total cells (left panel) and the cell fraction in blood (middle panel) and MPE (right panel) for each patient. **g**, Visualization of T cell receptor (TCR) clonotypes both identified in Cytotoxic CD8$^+$ T cells and Exhausted CD8$^+$ T cells. Left panel: tSNE plots of

the Cytotoxic $CD8^+$ T cells and Exhausted $CD8^+$ T cells. Shared TCR means TCR shared in Cytotoxic $CD8^+$ T cells and Exhausted $CD8^+$ T cells. Right panel: Fraction of the cell TCR status in Cytotoxic $CD8^+$ T cells and Exhausted $CD8^+$ T cells. **h**, Violin plot of exhausted score for each cells cluster in $CD8^+$ T cells.

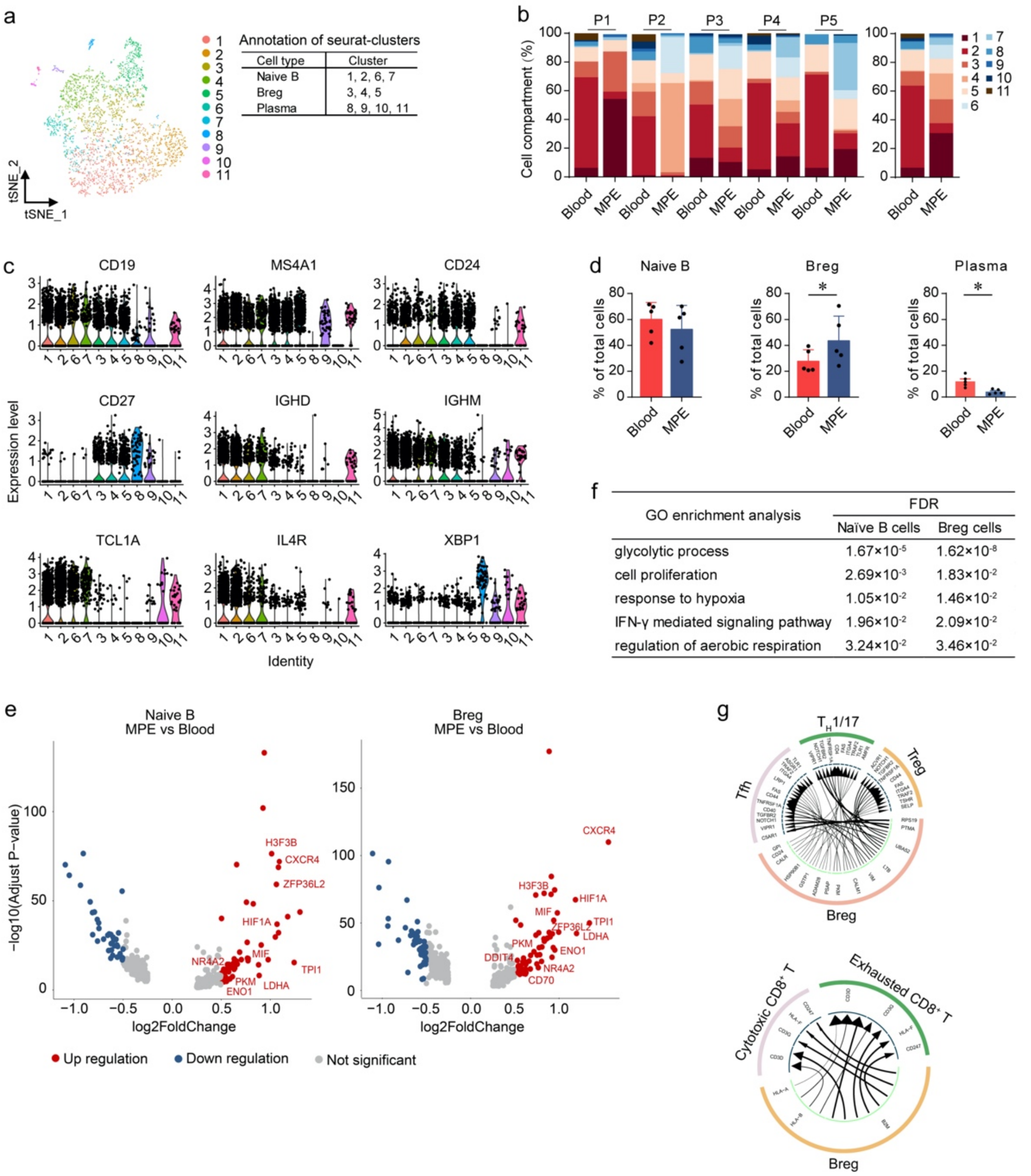

f

GO enrichment analysis	FDR	
	Naïve B cells	Breg cells
glycolytic process	1.67×10^{-5}	1.62×10^{-8}
cell proliferation	2.69×10^{-3}	1.83×10^{-2}
response to hypoxia	1.05×10^{-2}	1.46×10^{-2}
IFN-γ mediated signaling pathway	1.96×10^{-2}	2.09×10^{-2}
regulation of aerobic respiration	3.24×10^{-2}	3.46×10^{-2}

Supplementary Fig. 3. Single-cell transcriptional profiling of B cells from MPE and blood. a, tSNE plots of B cells and annotation of seurat-clusters with cell subtypes. **b**, Average proportion of each seurat-cluster derived from each patient (left panel), and MPE or blood (right panel). **c**, Violin plot of canonical marker genes for B cells in each cell subset. X-axis: seurat-clusters in Figure 3a. **d**, Frequencies of B cells types in MPE and blood according to the t-SNE plot using scRNA-seq data. Data are presented as mean ± SD. Comparisons were made using two tailed paired Student's *t*-test. Blood, n = 5 samples, MPE, n = 5 samples. **e**, Scatter plot of differently expressed genes of the MPE and blood in Naïve B cells (left panel) and Bregs (right panel). **f**, GO enrichment analysis of differentially express genes between MPE and blood samples in Breg cells or Naïve B cells. The significance was by Fisher's exact test and adjusted by Benjamini-Hochberg correction. **g**, The predicted interactions of Breg cells and $CD4^+$ T cells ($T_H1/17$, Treg and Tfh; left panel) or $CD8^+$ T cells (cytotoxic $CD8^+$ T and exhausted $CD8^+$ T; right panel) mediated by ligand and receptor.

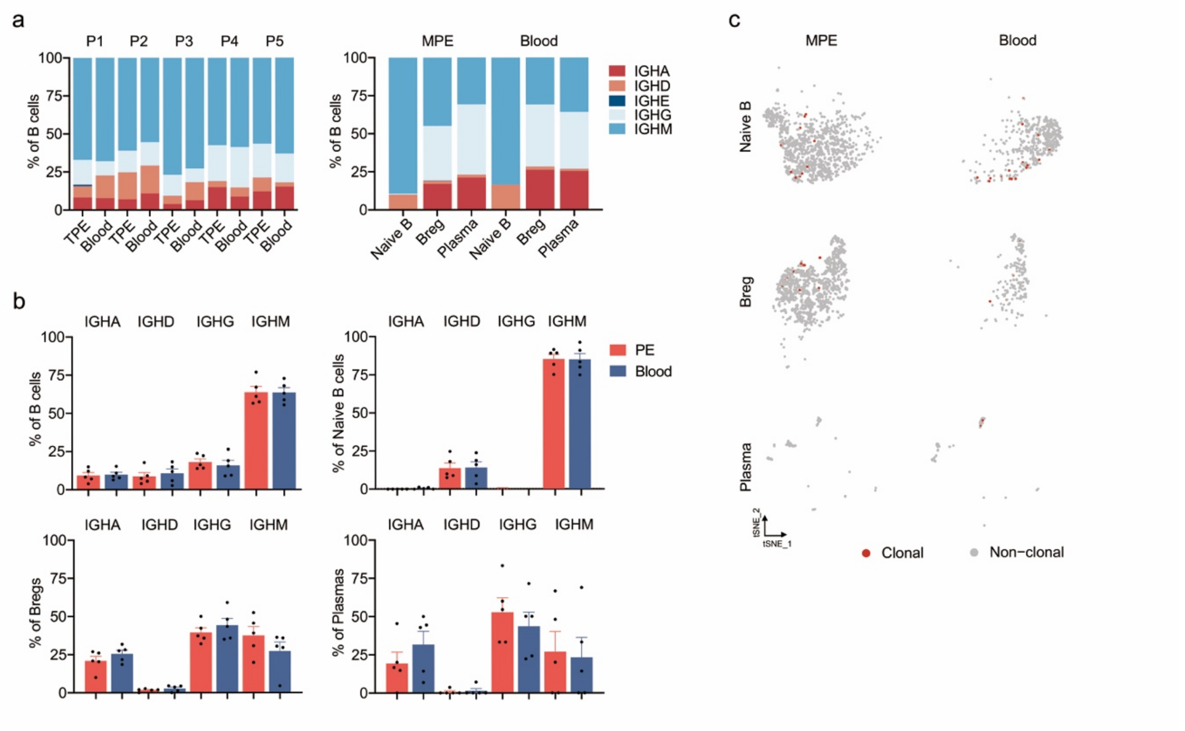

Supplementary Fig. 4. Clonal expansion of B cells defined by BCR. a, Fraction of the Ig isotype (IGHA, IGHD, IGHG and IHGM) fraction of each patient (left panel) or cell cluster (right panel). **b**, Frequencies of Ig isotype of total B cells and each cluster in MPE and blood (Data are presented as mean ± SD. Comparisons were made using two tailed paired Student's *t*-test, all $P > 0.05$. Blood, n = 5 samples, PE, n = 5 samples). **c**, The tSNE plot shows the BCR expansion status in MPE and blood.

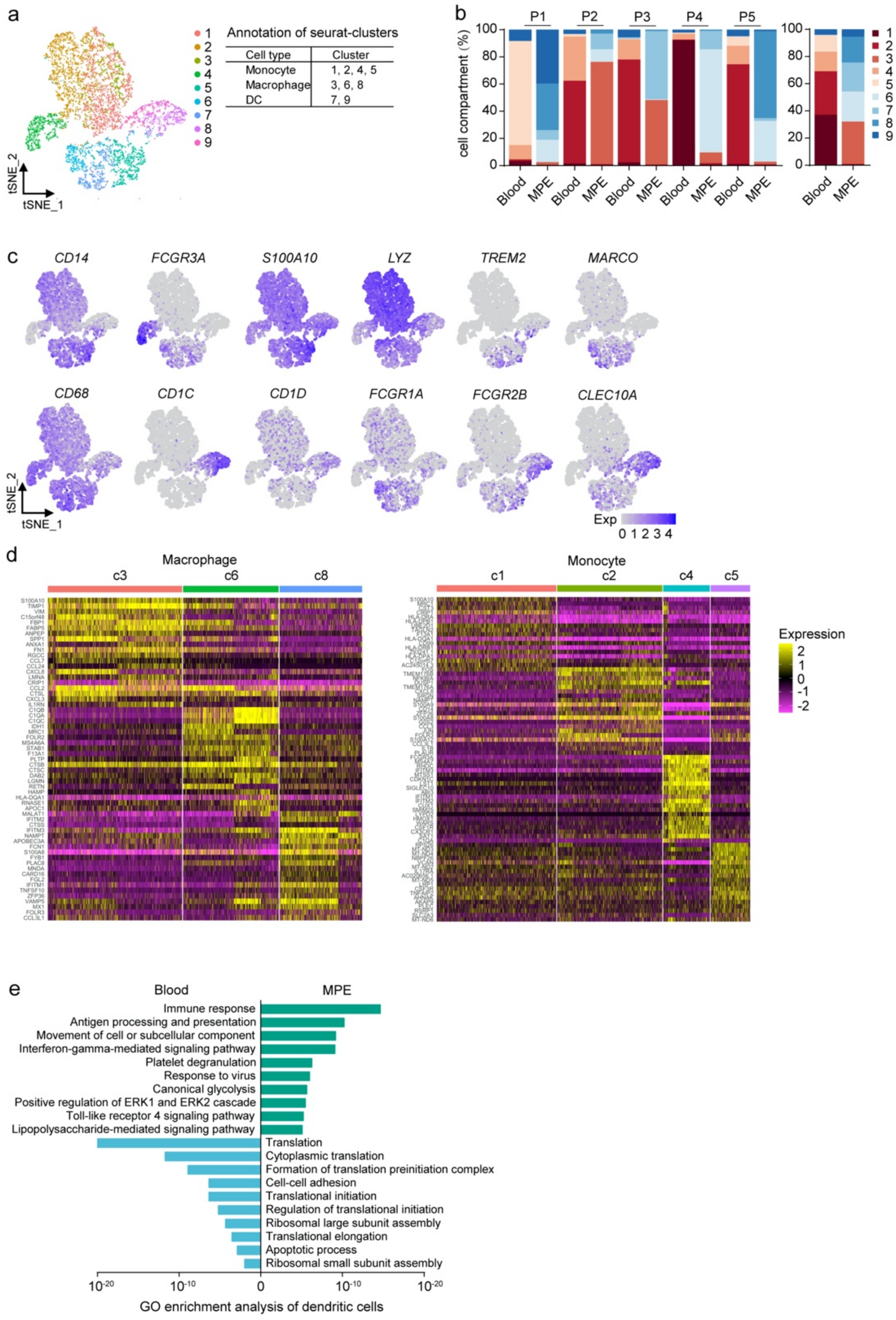

Supplementary Fig. 5. Single-cell transcriptional profiling of myeloid cells from MPE and blood. a, tSNE plots of Myeloids and annotation of seurat-clusters with cell subtypes. **b**, Average proportion of each seurat-cluster derived from each patient (left panel), and MPE or blood (right panel). **c**, Canonical cell markers of myeloid cells. Data are colored according to expression levels. **d**, Heatmaps of differentially express genes in monocyte (left panel) or macrophage (right panel). **e**, GO enrichment analysis of differentially express genes between MPE and blood samples in dendritic cell subsets. The significance was by Fisher's exact test and adjusted by Benjamini-Hochberg correction.

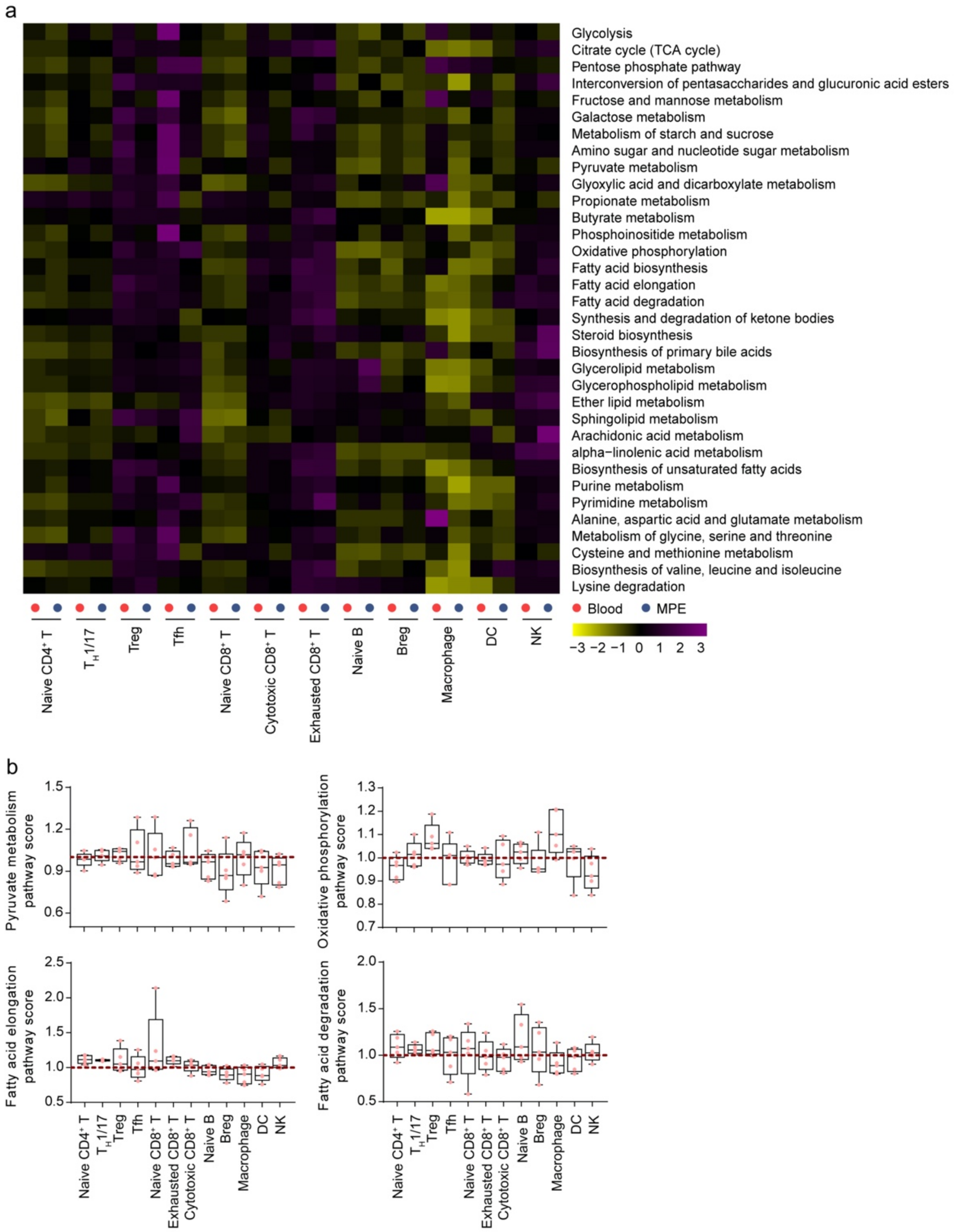

Supplementary Fig. 6. Metabolic heterogeneity in MPE and blood. a, Heatmap of the average enrichment score of the indicated metabolic pathways in in MPE and blood immune cell subtypes. **b**, Distributions of average enrichment score of the indicated metabolic pathways in MPE immune cell subtypes. n = 5 samples. The box plots were defined by the interquartile range (IQR, the range between the 25% and 75%) and the median, whiskers represent the upper and lower value within 1.5 times the IQR.

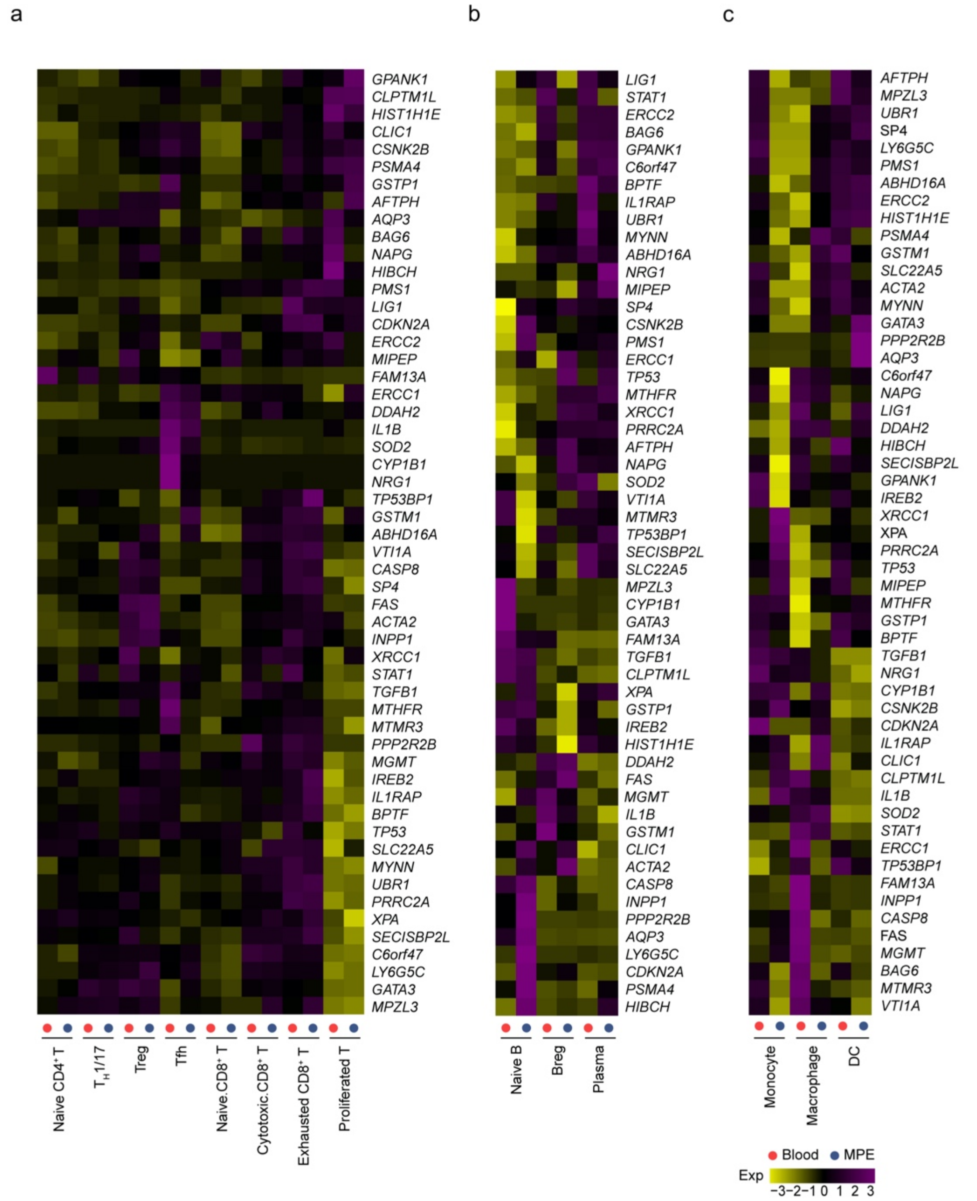

Supplementary Fig. 7. Cell-type specificity of genes associated with non-small cell lung cancer susceptibility in immune cells from MPE and blood using scRNA-seq. The heatmaps show the average expression of genes previously indicated in genome-wide association studies of non-small cell lung cancer across (**a**) T cell subsets, (**b**) B cell subsets and (**c**) myeloid cell subsets.

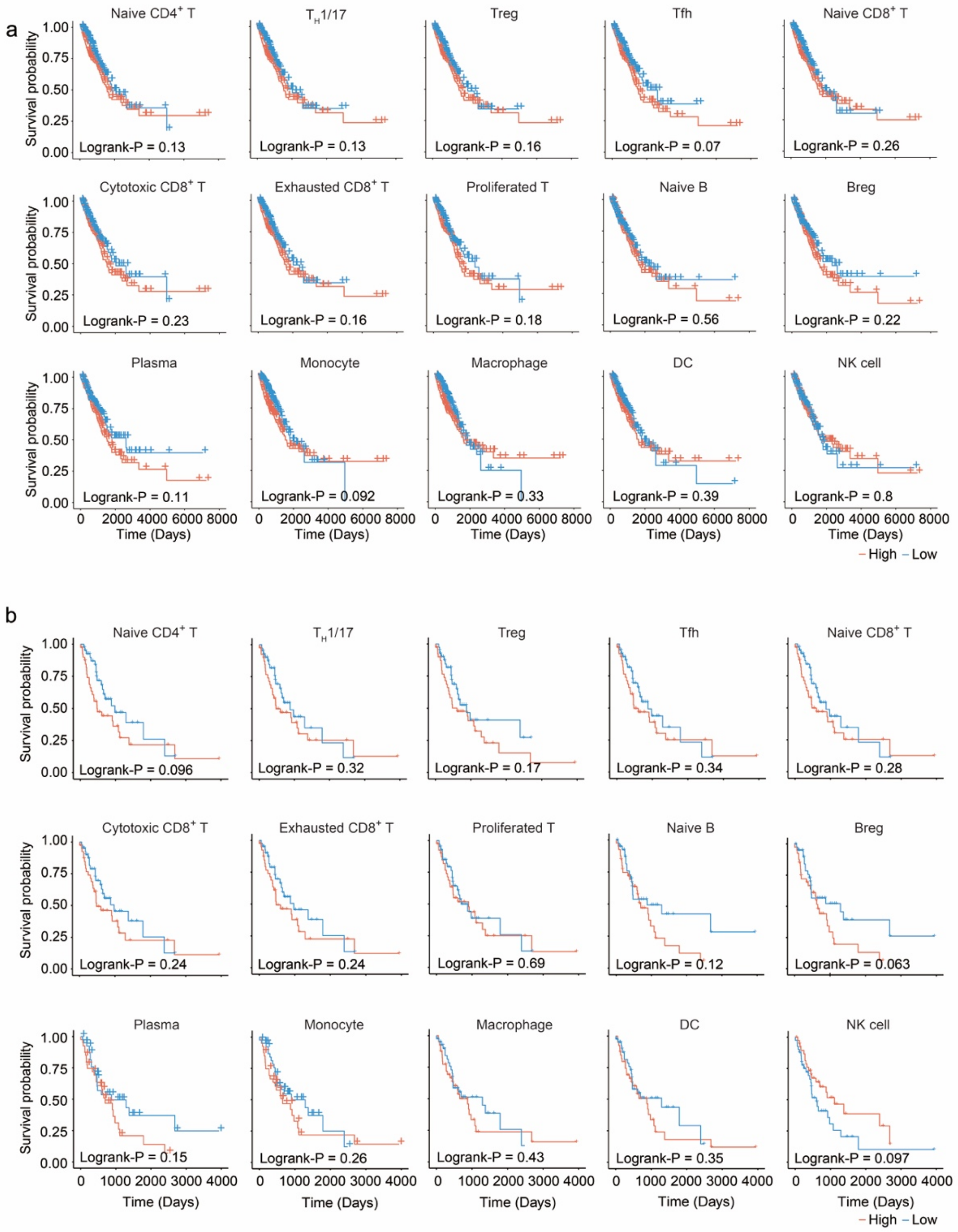

Supplementary Fig. 8. Survival analysis for signature genes of 15 cell types in The TCGA LUAD data. The Kaplan-Meier overall survival curves of TCGA LUAD patients (**a**) or TCGA LUAD patients with stage III and IV (**b**) grouped by the gene signature of 15 cell types. The high and low group are divided by the median value of mean expression of signature gene after normalization by CIBERSORT. The significant was calculated using the two-sided log-rank test.

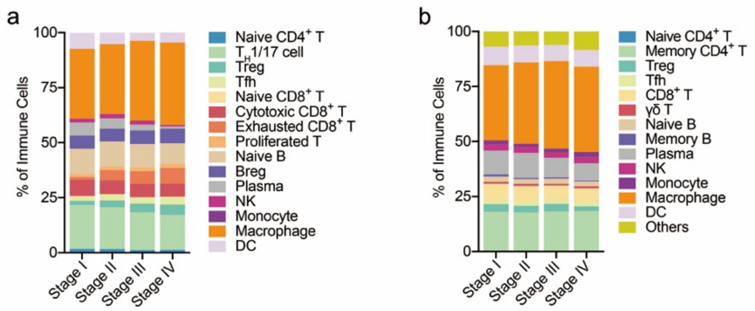

Supplementary Fig. 9. Proportion of cell types in TCGA LUAD data. a, Proportion analysis in TCGA LUAD data for 15 cell types identified in MPE. **b**, Proportion analysis in TCGA LUAD data for CIBERSORT cell type.

Supplementary Table 1. Metric information for five MPE patients induced by lung adenocarcinoma

Patient ID	Sex	Age (yr)	Sample Type	Reads	Genes	Mean Reads per Cell	Median Genes per Cell	Median UMIs per Cell	Cells (before filtering)	Cells (After filtering)
P1	Male	68	Blood	1,037,825,378	20,068	152,598	1,560	3,807	6,801	5,793
			MPE	852,337,548	19,782	190,296	1,560	4,307	4,479	4,282
P2	Female	64	Blood	800,604,289	19,671	138,921	1,416	3,525	5,763	3,517
			MPE	849,787,038	20,998	143,109	1,805	4,396	5,938	5,609
P3	Male	65	Blood	875,444,680	20,930	106,592	1,752	4,356	8,213	7,460
			MPE	853,575,131	21,707	103,551	1,549	4,668	8,243	7,650
P4	Male	72	Blood	902,217,037	19,956	107,330	1,580	4,223	8,406	7,775
			MPE	849,451,420	21,134	97,964	1,588	4,573	8,671	8,329
P5	Male	56	Blood	805,098,625	20,080	154,737	1,686	4,721	5,203	4,748
			MPE	898,216,524	21,590	116,788	1,660	4,640	7,691	7,219

MPE = malignant pleural effusion.

Supplementary Table 2. Gene list in genome-wide association studies related analysis

Gene	
CLPTM1L	IREB2
TP53	PSMA4
GSTP1	MIPEP
GSTM1	SLC22A5
ERCC2	BPTF
ERCC1	PRRC2A
XPA	HIST1H1E
CYP1B1	VTI1A
IL1B	NAPG
XRCC1	HIBCH
CASP8	INPP1
MTHFR	PMS1
FAS	STAT1
SOD2	SP4
MGMT	MYNN
TGFB1	AFTPH
LIG1	AQP3
IL1RAP	NRG1
FAM13A	CDKN2A
ACTA2	MPZL3
MTMR3	SECISBP2L
GATA3	LY6G5C
PPP2R2B	ABHD16A
BAG6	CLIC1
C6orf47	DDAH2
CSNK2B	UBR1
GPANK1	TP53BP1
