# Supplementary material for: Single-cell analysis of diverse immune phenotypes in malignant pleural effusion
Source: Nat Commun. 2021 Nov 18;12:6690. doi: 10.1038/s41467-021-27026-9 (PMC8602344; doi:10.1038/s41467-021-27026-9)
Supplement: Supplementary file 2 — Description of Additional Supplementary Files [file 41467_2021_27026_MOESM2_ESM.pdf]

Description of Additional Supplementary Files

File Name: Supplementary Data 1
Description: The differentially expressed genes of each cell type between malignant pleural effusion and blood.

File Name: Supplementary Data 2
Description: Metabolism pathway signature genes downloaded from KEGG.
