# Supplementary material for: Single-cell analysis of diverse immune phenotypes in malignant pleural effusion
Source: Nat Commun. 2021 Nov 18;12:6690. doi: 10.1038/s41467-021-27026-9 (PMC8602344; doi:10.1038/s41467-021-27026-9)
Supplement: Supplementary file 5 — Reporting Summary [file 41467_2021_27026_MOESM5_ESM.pdf]

nature portfolio

Corresponding author(s): Kan Zhai, Huan-Zhong Shi

Last updated by author(s): Oct 1, 2021

Reporting Summary

Nature Portfolio wishes to improve the reproducibility of the work that we publish. This form provides structure for consistency and transparency in reporting. For further information on Nature Portfolio policies, see our Editorial Policies and the Editorial Policy Checklist.

Statistics

For all statistical analyses, confirm that the following items are present in the figure legend, table legend, main text, or Methods section.

n/a	Confirmed	
☐	☒	The exact sample size (*n*) for each experimental group/condition, given as a discrete number and unit of measurement
☐	☒	A statement on whether measurements were taken from distinct samples or whether the same sample was measured repeatedly
☐	☒	The statistical test(s) used AND whether they are one- or two-sided<br>*Only common tests should be described solely by name; describe more complex techniques in the Methods section.*
☐	☒	A description of all covariates tested
☐	☒	A description of any assumptions or corrections, such as tests of normality and adjustment for multiple comparisons
☐	☒	A full description of the statistical parameters including central tendency (e.g. means) or other basic estimates (e.g. regression coefficient) AND variation (e.g. standard deviation) or associated estimates of uncertainty (e.g. confidence intervals)
☐	☒	For null hypothesis testing, the test statistic (e.g. *F*, *t*, *r*) with confidence intervals, effect sizes, degrees of freedom and *P* value noted<br>*Give P values as exact values whenever suitable.*
☒	☐	For Bayesian analysis, information on the choice of priors and Markov chain Monte Carlo settings
☒	☐	For hierarchical and complex designs, identification of the appropriate level for tests and full reporting of outcomes
☒	☐	Estimates of effect sizes (e.g. Cohen's *d*, Pearson's *r*), indicating how they were calculated

Our web collection on statistics for biologists contains articles on many of the points above.

Software and code

Policy information about availability of computer code

Data collection	Single cell RNA sequencing of five paired MPE and blood samples was perform by 10x Genomics (Pleasanton, CA) and NovaSeq 6000 System (Illumina, San Diego, CA) with a pair-end 150 bp reading strategy.
Data analysis	Data analysis was performed by Cell Ranger (v.3.0.2) and R software (v.3.5.3) (package: Seurat (v.3.0.0), Monocle 3, GO.db (3.11.4), ggplot2 (3.3.3), Destiny (v.2.6.2), ggsci (2.9), survival (3.2-10) and survminer (0.4.9)), CellPhoneDB (www.CellPhoneDB.org) and CIBERSORT (v1.03).

For manuscripts utilizing custom algorithms or software that are central to the research but not yet described in published literature, software must be made available to editors and reviewers. We strongly encourage code deposition in a community repository (e.g. GitHub). See the Nature Portfolio guidelines for submitting code & software for further information.

Data

Policy information about availability of data

All manuscripts must include a data availability statement. This statement should provide the following information, where applicable:

- Accession codes, unique identifiers, or web links for publicly available datasets
- A description of any restrictions on data availability
- For clinical datasets or third party data, please ensure that the statement adheres to our policy

The raw sequence data and partially processed data have been deposited in Genome Sequence Archive for Human (HRA000153) and and Gene Expression Omnibus database (GSE185058).

Field-specific reporting

Please select the one below that is the best fit for your research. If you are not sure, read the appropriate sections before making your selection.

☒ Life sciences ☐ Behavioural & social sciences ☐ Ecological, evolutionary & environmental sciences

For a reference copy of the document with all sections, see nature.com/documents/nr-reporting-summary-flat.pdf

Life sciences study design

All studies must disclose on these points even when the disclosure is negative.

Sample size	Single cell RNA sequencing was performed on 10 Samples (5 paired MPE and peripheral blood samples), and 220G of sequencing data and 10 G of TCR/BCR sequencing data was obtained from each sample, which was sufficient to support our landscape study.
Data exclusions	Genes with an expression ratio of < 0.1% and cells with less than 200 or more than 6,000 detected genes were removed. Low-quality cells with more than 10% unique molecular identifiers derived from the mitochondrial genome and doublets identified by DoubletFinder were removed.
Replication	Biological replication of the experiment was not involved in this study.
Randomization	Artificial grouping was not involved in this study.
Blinding	NA

Reporting for specific materials, systems and methods

We require information from authors about some types of materials, experimental systems and methods used in many studies. Here, indicate whether each material, system or method listed is relevant to your study. If you are not sure if a list item applies to your research, read the appropriate section before selecting a response.

Materials & experimental systems

n/a	Involved in the study	
☒	☐	Antibodies
☒	☐	Eukaryotic cell lines
☒	☐	Palaeontology and archaeology
☒	☐	Animals and other organisms
☐	☒	Human research participants
☒	☐	Clinical data
☒	☐	Dual use research of concern

Methods

n/a	Involved in the study	
☒	☐	ChIP-seq
☒	☐	Flow cytometry
☒	☐	MRI-based neuroimaging

Human research participants

Policy information about studies involving human research participants

Population characteristics	Five patients with definite diagnosis of MPE (4 males and 1 female; mean age, 65 years old, range, 56 – 72 years old) were enrolled in our study.
Recruitment	Patients with definite diagnosis of pleural effusions were enrolled in our study, and they all signed the informed consent according to the approved guideline. A diagnosis of MPE was established by the appearance of malignant cells in pleural effusion and/or on closed pleural biopsy samples. Patients included in the study were with no history of corticosteroids usage, anti-tumor therapy, or any other drugs known to affect the immunological condition.
Ethics oversight	This study was conducted in accordance with the approved guidelines of the Institutional Review Boards of Beijing Chao-Yang Hospital, Capital Medical University (No. 2018-ke-327); Union Hospital, Tongji Medical College (No.[2019]S879); and Nanning Fourth People's Hospital (No. [2019]28).

Note that full information on the approval of the study protocol must also be provided in the manuscript.
